# Emergency Operation Scheme Generation for Urban Rail Transit Train Door Systems Using Retrieval-Augmented Large Language Models

**DOI:** 10.3390/s26062006

**Published:** 2026-03-23

**Authors:** Lu Huang, Zhigang Liu, Chengcheng Yu, Tianliang Zhu, Bing Yan

**Affiliations:** 1 School of Urban Railway Transportation, Shanghai University of Engineering Science, Shanghai 201620, China; luhuang@sues.edu.cn (L.H.);; 2 College of Transportation, Tongji University, Shanghai 200092, Chinazhutianliang@tongji.edu.cn (T.Z.)

**Keywords:** urban rail transit, train door faults, incident response, knowledge base, retrieval and reranking, human–machine collaboration, safety-critical decision support

## Abstract

Urban rail transit (URT) train-door failures are safety-critical and can cause cascading service disruptions, yet existing emergency operation schemes (EOSs) are often static, difficult to adapt to evolving fault patterns, and hard to verify against updated regulations. This study proposes a retrieval-augmented large language model (LLM) framework for executable and evidence-traceable EOS generation. Multi-source heterogeneous incident evidence (structured work orders, operational impact records, and unstructured maintenance/dispatch narratives) is normalized into a structured incident representation, and a hybrid retriever (dense + BM25) with cross-encoder reranking selects compact regulatory clauses and historical cases under a fixed context budget. The generator is fine-tuned with structured objectives to enforce schema compliance, role assignment, and citation grounding. Experiments on 776 passenger-door incidents from Shanghai URT (2019–2024) show that Hybrid + rerank achieves the best retrieval quality (Recall@5 = 0.78; Coverage@*B* = 0.71; FirstHit/*B* = 0.46). For generation, the full setting improves operational usability, reaching SchemaPass = 0.88, RoleAcc = 0.91, CiteCov = 0.73, and UsableAns = 0.83, compared with 0.15 UsableAns for a pure LLM baseline and 0.26 for prompting with RAG only. These results indicate that combining high-utility retrieval with structure- and citation-aware fine-tuning substantially improves the executability and verifiability of safety-critical operation schemes.

## 1. Introduction

Urban rail transit (URT) systems play a critical role in the transportation infrastructure of modern cities, providing a reliable, efficient, and environmentally friendly means of travel [1,2,3,4]. Among the various components of a URT train, the door system is particularly vital, ensuring the safety of passengers, facilitating the flow of people in and out of the train, and maintaining operational reliability [5]. However, since the train door system directly interacts with passengers, it is one of the most prone components to malfunction. Failures in this system can lead to significant disruptions, safety concerns, and service delays. For example, a failure in the door mechanism can cause extended boarding times, delays in the train’s departure, or even safety hazards [6,7], such as doors not opening properly during emergency evacuations [8]. These issues not only affect passengers but also strain the operational efficiency of the entire urban rail network. To mitigate the operational risks, an effective emergency operation scheme (EOS) is essential. EOS refers to a set of predefined actions taken to handle train door failures in a way that minimizes operational disruptions, restores normal operations swiftly, and ensures passenger safety [9,10]. Traditional methods of handling URT train door failures often rely on expert systems, manual checks, and fixed response protocols, which may not be optimal for every failure scenario. As a result, there is a growing need for adaptive, data-driven methods to improve the efficiency and flexibility of emergency operations [11,12].

In current URT practice, train-door failure handling is primarily a human-coordinated operational process rather than an automatic control process. When a door fault occurs, frontline personnel such as the driver, OCC/dispatch staff, station staff, and maintenance personnel follow predefined emergency procedures, operating manuals, and training requirements to assess the fault, isolate the affected door when necessary, maintain service safety, and organize subsequent maintenance actions. The practical difficulty is therefore not to autonomously open or close the door through an AI controller, but to help responsible personnel identify a timely, role-consistent, and regulation-compliant response sequence under operational time pressure.

Recent advancements in large language models (LLMs) and retrieval-augmented generation (RAG) techniques [13,14] offer promising opportunities to enhance fault detection, response strategies, and decision-making in emergency scenarios. For instance, failure and maintenance logs, often in the form of text, can be challenging to process using traditional deep learning models, which typically focus on tabular/structured data [15]. However, generating an effective EOS requires understanding the primary causes of faults and applying the optimal solution for each cause to minimize the operational impact [16]. Integrating LLMs, which are capable of understanding and processing textual data, with structured fault-related data (such as the tabled failure causes) offers a more efficient and dynamic way to generate emergency responses. Current EOS implementation still has three limitations: (1) inadequate integration of heterogeneous operational records with historical maintenance knowledge, (2) limited adaptability to newly emerging failure patterns, and (3) insufficient validation mechanisms for generated solutions against safety regulations. To address these gaps, this study proposes an LLM-based framework enhanced by retrieval-augmented generation (RAG) for EOS generation in URT train-door failures. We construct a domain-specific knowledge base by integrating multi-source operational information, including structured work orders, dispatch/operations records, and unstructured maintenance logs and fault narratives. These processed data are transformed into domain-specific failure-scenario–solution pairs for model adaptation and evidence-grounded generation.

Therefore, this study makes four technical contributions beyond the generic use of LLMs with retrieval as follows:

(1) First, we formulate EOS generation for URT train-door failures as a safety-critical, retrieval-augmented conditional generation task, rather than a general question-answering or recommendation problem.
(2) Second, we construct a domain-specific evidence pipeline that jointly uses structured operational fields, unstructured maintenance/dispatch narratives, regulations, standard operating procedures, canonicalized historical cases, and a maintenance knowledge-graph channel to support context-aware generation.
(3) Third, we introduce structure-aware generation controls, including schema-constrained serialization, explicit role assignment, citation grounding, and compliance-aware decoding, so that the generated EOS is executable and traceable rather than merely fluent.
(4) Fourth, we validate the framework on 776 real train-door incidents using both retrieval-oriented and operational-usability-oriented metrics, together with expert evaluation, to assess whether the generated schemes are practically usable in URT operations.

The remainder of this study is organized as follows. Section 2 reviews the existing studies. Section 3 presents the methodology framework. In Section 4, we introduce the data sources, and Section 5 presents the result analysis. And Section 6 presents the discussion of the results.  Section 7 concludes this study and points out the future research direction.

## 2. Literature Review

### 2.1. Emergency Operation Schemes (EOSs) in Urban Rail Transit Systems

The effective EOSs are critical for managing safety risks and hence minimizing operational disruptions in URT systems. EOS refers to predefined procedures and actions that are triggered when a fault occurs, aiming to restore normal operations as swiftly as possible while ensuring the safety of passengers [17]. The typical emergency response process in URT systems not only defines how different department performs their tasks but also entails close collaboration and interaction between various teams [18]. According to a review of 52 EOSs for China’s URT projects, effective EOSs include seven components: information reporting, initial response, activation of emergency operations, emergency rescue, information dissemination and news reporting, safety protection for emergency personnel and the public, and the end of emergency response [19]. These components are common practices across different URT systems, forming a comprehensive framework for addressing failures in real-time.

Ding et al. [16] proposed a data-driven approach to enhance urban rail transit safety management by developing a Dispatching Fault Log Management and Analysis Database System (DFLMIS). They analyzed over 110,000 operational fault logs from Shanghai Metro (2011–2015) using data mining techniques [20], identifying 21,156 risk sources across seven primary fault categories, including vehicle failures, communication signals, and passenger-related factors. Zhang et al. [21] developed a metro operation incident database (MOID) and compiled incident data to identify 24 critical accident precursors through incident types, causes, temporal patterns, and severity levels, supported by a proposed four-pillar organizational structure involving supervision, research, implementation, and manufacturer collaboration. Yu et al. [22] identified typical faults in platform door insulation, particularly the risk of electric shock caused by passenger contact, and evaluated the underlying causes of compromised insulation integrity.

Regarding fault analysis methods in URT systems, Nguyen et al. [23] developed a safety risk assessment framework for Vietnam’s inaugural light rail transit line (UMRT Line HN2A) by applying fault tree analysis (FTA) to address gaps in conventional risk evaluation methods for aging infrastructure. Their method integrates theoretical risk modeling with real-world fault data and establishes a dynamic framework in which continuous data collection refines risk probabilities. Recent studies have extended URT fault analysis to specific subsystems, particularly door systems as critical components. For example, Chen et al. [24] introduced a Bayesian network (BN)-based approach for vehicle door fault diagnosis, addressing uncertainties in failure patterns through probabilistic reasoning. This data-driven BN model demonstrated 92% diagnostic accuracy in simulations and provided real-time maintenance recommendations, representing a substantial advance over traditional threshold-based alarm systems. Complementing this probabilistic approach, Louadah et al. [25] applied FTA to intercity train door reliability by constructing data-driven fault trees to quantify subsystem failure contributions. Their framework further incorporated maintenance records and operational downtime costs, revealing that 68% of door-related service interruptions originated from electromechanical actuator failures. This implementation enabled comparative reliability assessments of alternative train door designs and provided actionable insights for both engineering optimization (e.g., seal mechanism redundancy) and maintenance scheduling (e.g., sensor calibration intervals).

Beyond equipment-level fault diagnosis and maintenance analysis, recent studies have also used metro smart card data to quantify passenger-facing operational impacts under uncertainty and disruption. For example, Zhang et al. [26] developed a dynamic accessibility framework based on metro smart card data and showed that accessibility varies significantly across time periods and can be overestimated if travel time uncertainty is ignored. Liu et al. [27] proposed an AFC-based framework to evaluate unplanned metro disruptions from both system-performance and passenger-response perspectives, while Mo et al. [28] inferred multiple passenger response behaviors under rail disruptions using smart card transaction data. Liu et al. [29] further modeled the duration of disruption impacts on passenger trips and showed that passenger impacts may persist longer than the train delay itself. Although these studies do not directly generate emergency operation schemes, they are highly relevant to the present study because they demonstrate how metro disruption severity, passenger delay propagation, and service degradation can be quantified from operational travel data. This perspective complements maintenance- and fault-oriented EOS research by highlighting the passenger and system-performance consequences that emergency handling strategies are ultimately intended to mitigate.

### 2.2. LLM and RAG Techniques in EOS Generation

The use of LLM and RAG techniques in the context of EOS has recently gained significant attention, particularly for addressing the challenges posed by complex, real-time decision-making in URT systems. LLMs, such as GPT and BERT, are designed to understand and process natural language, making them well-suited for handling unstructured data like maintenance logs, fault reports, and failure history, which are often crucial in diagnosing and responding to incidents in transportation systems [30]. In the context of EOS, these models can be utilized to understand the context of faults and generate relevant, accurate emergency response actions based on both historical and real-time data.

RAG techniques combine the generative power of LLMs with external knowledge retrieval, significantly enhancing the ability of these models to adapt to new and evolving scenarios. RAG operates by retrieving information from large external databases or documents before generating a response, ensuring that the output is not only informed by historical data but also reflects the latest updates in safety regulations, operational data, and failure patterns [14]. This makes the model more adaptive and capable of handling evolving scenarios. For example, if a new failure pattern emerges in the door system of a URT train, RAG can retrieve the most relevant past cases, real-time data, and updated safety protocols to generate an accurate emergency response.

In terms of EOS generation, recent studies have shown how LLMs and retrieval-augmented generation (RAG) can be used in crisis management and transportation system optimization. Otal et al. [31] proposed an LLM-driven crisis management framework based on the open-source Llama2 model to enhance emergency response through real-time multimodal data analysis and public collaboration. In the URT research area, Chen et al. [32] introduced a framework called DelayPTC-LLM, which leverages LLMs to predict passenger travel choices during train delays. This framework has addressed challenges like data sparsity and sample imbalance, common in rare delay events, which uses context-aware prompt engineering, structuring the LLM inputs to capture passenger behavior (such as tolerance thresholds for delays) and delay-specific features (like severity and propagation across the network). This approach integrates structured operational data into textual prompts, allowing LLMs to infer passenger behavior from sparse datasets using few-shot learning techniques. While there has been limited direct research on the application of LLMs and RAG techniques for EOS generation in URT systems, the methodologies developed for integrating structured data (such as operational logs and sensor data) with unstructured data can be applied to design more effective EOS frameworks. By leveraging LLMs and RAG, URT systems are capable of developing dynamic, context-sensitive emergency response systems that are always updated based on real-time data and evolving failure patterns.

In addition to retrieval grounding, a closely related recent research direction focuses on making LLM outputs reliably structured rather than purely free-form. Grammar-constrained decoding has been shown to enforce task-specific formal output structures and improve performance on structured NLP tasks without task-specific fine-tuning [33]. Related work has further demonstrated that regular expressions and context-free grammars can be compiled into efficient guided decoding procedures, making schema-constrained generation practical for real applications [34]. Other studies frame prompting and constrained generation in a more declarative manner, for example by treating prompting as a programmable interface with explicit constraints and control flow over model outputs [35]. More recent advances have also extended constrained decoding to low-overhead subword-aligned decoding [36], black-box settings without logit access [37], and JSON-schema-oriented structured generation [38].

This line of research is directly relevant to EOS generation in URT systems, because the target output is not open-ended text but a standardized operational procedure that must satisfy fixed structural requirements. However, most prior constrained-generation studies evaluate general structured prediction or information extraction tasks, rather than safety-critical operational text generation in which executability, responsibility allocation, and traceability to supporting evidence are all required simultaneously. This motivates the present study, which combines RAG-based evidence retrieval with structure-aware fine-tuning and compliance-sensitive decoding for EOS generation.

### 2.3. Research Gap

While substantial progress has been made in enhancing EOSs for urban rail transit systems, there remain several critical gaps that need to be addressed for these systems to fully benefit from modern technologies, such as LLMs and RAG. Current EOS implementations often rely on static, expert-based protocols that cannot effectively integrate real-time data with historical maintenance logs and operational records. This limits the ability to respond dynamically to failures as they arise. In many cases, real-time data from sensors, which can provide vital information about the train’s current operational state, is not adequately linked with historical fault records, reducing the efficiency of the system in identifying and addressing issues promptly. Recent studies have shown that integrating structured data (like sensor data) with unstructured data (like maintenance logs) can improve fault detection and diagnosis, yet this remains a challenge for existing EOS models.

Traditional EOS implementations operate on a fixed-response protocol, which may not be able to handle novel or evolving failure scenarios. For example, train door systems, which are highly complex and may exhibit different failure patterns over time, require a dynamic response system that can adapt to these changes. Current systems often lack the flexibility to adjust to new failure modes, which means that when unforeseen issues occur, the response may be suboptimal or delayed. This gap in adaptability is particularly relevant as URT systems become increasingly complex and data-rich. In addition, the ability to dynamically retrieve the most relevant safety regulations and incorporate them into the EOS generation process is a crucial need, which can be addressed through the integration of RAG techniques with LLMs.

These research gaps highlight the need for more advanced, adaptive, and data-driven EOS frameworks for URT systems. By leveraging LLMs and RAG techniques, it is possible to create an EOS that continuously integrates real-time operational data, adapts to failure scenarios, and ensures compliance with the latest safety standards.

## 3. Methodology

### 3.1. Research Framework

**Figure 1:**
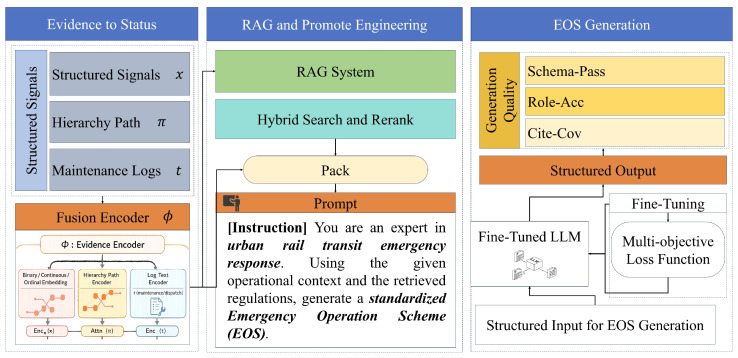
Overall architecture of the proposed RAG-enhanced EOS generation framework for URT train-door failures.

This study formulates emergency operation scheme (EOS) generation for urban rail train-door failures as a retrieval-augmented conditional generation problem, where heterogeneous operational evidence is first normalized into a unified representation and then used to construct question–answer supervision and an evidence index for retrieval as shown in Figure 1. Let N∈N denote the number of validated door-failure events after preprocessing, and let i∈{1,…,N} index an event. Each event is observed as a tuple $E_{i}=\left(x_{i}^{\left(b\right)},x_{i}^{\left(c\right)},x_{i}^{\left(o\right)},\pi_{i},t_{i}\right)$. Here, $x_{i}^{\left(b\right)}\in {\left\{0,1\right\}}^{d_{b}}$ is a binary feature vector of dimension $d_{b}$, $x_{i}^{\left(c\right)}\in R^{d_{c}}$ is a continuous feature vector of dimension $d_{c}$, $x_{i}^{\left(o\right)}\in {\left\{0,1,\ldots \right\}}^{d_{o}}$ is an ordinal/categorical feature vector of dimension $d_{o}$, $\pi_{i}$ is a hierarchical fault path in a component taxonomy, and $t_{i}\in \Sigma^{*}$ is an unstructured textual log over token alphabet Σ (with $\Sigma^{*}$ denoting all finite token sequences). The methodological objective is to learn a model that maps the event into an EOS text $a\in \Sigma^{*}$ that is executable and evidence-traceable.

A core challenge is that the features are mixed-type and partially hierarchical, which motivates a unified latent “failure state” representation. We define a fusion encoder Φ(·) that produces a vector $z_{i}\in R^{d_{z}}$ of dimension $d_{z}$,

(1) $$z_{i}=\Phi \left(x_{i}^{\left(b\right)},x_{i}^{\left(c\right)},x_{i}^{\left(o\right)},\pi_{i}\right).$$

To stabilize scale, continuous variables are standardized using dataset statistics. Let $\mu \in R^{d_{c}}$ and $\sigma \in R^{d_{c}}$ be the empirical mean and standard deviation, respectively, and define element-wise normalization

(2) $${\tilde{x}}_{i}^{\left(c\right)}=\left(x_{i}^{\left(c\right)}-\mu \right)⊘\sigma ,$$

where ⊘ denotes element-wise division. Ordinal/categorical variables are embedded to avoid brittle one-hot sparsity. Let $K_{j}\in N$ be the number of discrete values for the *j*-th ordinal/categorical field, and let ${\mathrm{Emb}}_{j}:\left\{0,\ldots ,K_{j}-1\right\}\to R^{d_{e}}$ be an embedding map with embedding dimension $d_{e}$. Writing $x_{i,j}^{\left(o\right)}$ for the *j*-th component of $x_{i}^{\left(o\right)}$, we define the concatenated ordinal embedding

(3) $$e_{i}^{\left(o\right)}={∥}_{j=1}^{d_{o}}{\mathrm{Emb}}_{j}\left(x_{i,j}^{\left(o\right)}\right)\in R^{d_{o}d_{e}},$$

where ∥ denotes vector concatenation over fields. The hierarchical fault descriptor $\pi_{i}$ is modeled as a path in a rooted taxonomy tree T=(V,E), where V is the node set and E is the edge set. A path is written as $\pi_{i}=\left(v_{i}^{\left(1\right)},\ldots ,v_{i}^{\left(L\right)}\right)$, where L∈N is the taxonomy depth and $v_{i}^{\left(l\right)}\in V$ denotes the node at level *ℓ*. Let $E\in R^{\left|V\right|\times d_{h}}$ be a trainable embedding matrix with node-embedding dimension $d_{h}$, and define $h_{i}^{\left(l\right)}=E\left[v_{i}^{\left(l\right)}\right]\in R^{d_{h}}$. We aggregate the path by attention to capture which hierarchical level is most informative for EOS decisions. With attention hidden size $d_{a}\in N$, parameters $W\in R^{d_{a}\times d_{h}}$, $b\in R^{d_{a}}$, and $u\in R^{d_{a}}$, we compute

(4) $$\alpha_{i}^{\left(l\right)}=\frac{\exp\;\left(u^{⊤}\tanh\left(Wh_{i}^{\left(l\right)}+b\right)\right)}{\sum_{r=1}^{L}\exp\;\left(u^{⊤}\tanh\left(Wh_{i}^{\left(r\right)}+b\right)\right)},\;e_{i}^{\left(h\right)}=\sum_{l=1}^{L}\alpha_{i}^{\left(l\right)}h_{i}^{\left(l\right)}\in R^{d_{h}}.$$

The final fused state is obtained by applying a nonlinear projection. Let $W_{f}\in R^{d_{z}\times \left(d_{b}+d_{c}+d_{o}d_{e}+d_{h}\right)}$ and $b_{f}\in R^{d_{z}}$ be parameters, and let σ(·) be an element-wise nonlinearity (e.g., GELU). Then

(5) $$z_{i}=\sigma \left(W_{f}\left[x_{i}^{\left(b\right)}{\tilde{x}}_{i}^{\left(c\right)}e_{i}^{\left(o\right)}e_{i}^{\left(h\right)}\right]+b_{f}\right)\in R^{d_{z}}.$$

The framework then explicitly constructs the supervision signal as question–answer pairs, which is essential because EOS generation is evaluated as text generation conditioned on a normalized failure description rather than direct label prediction. We define a question synthesis operator $T_{Q}:\Sigma^{*}\times R^{d_{z}}\to \Sigma^{*}$ that converts raw log $t_{i}$ and latent state $z_{i}$ into a standardized natural-language “question” $q_{i}\in \Sigma^{*}$,

(6) $$q_{i}=T_{Q}\left(t_{i},z_{i}\right).$$

In practice, $T_{Q}$ is implemented as a deterministic slot-based template plus controlled summarization to preserve dispatch-critical entities; we formalize slot completeness to avoid under-specified training prompts. Let S be a finite set of required slots (e.g., line, station, train identifier, door index, symptom, severity, context flags) and let present(s,q)∈{0,1} indicate whether slot s∈S appears in question *q*. The normalized coverage score is

(7) $$C\left(q\right)=\frac{1}{\left|S\right|}\sum_{s\in S}\left[\mathrm{present}\left(s,q\right)=1\right]\in \left[0,1\right],$$

where [·] is the indicator function. Only questions with $C(q_{i})\geq \eta$ are retained, where η∈(0,1] is a predefined threshold. The corresponding “answer” $a_{i}\in \Sigma^{*}$ is the EOS, obtained by canonicalizing raw operational descriptions. Let $y_{i}\in \Sigma^{*}$ be an EOS narrative extracted from logs/manuals; a canonicalizer $T_{A}:\Sigma^{*}\to \Sigma^{*}$ produces

(8) $$a_{i}=T_{A}\left(y_{i}\right).$$

To reduce ambiguity and support downstream verification, we also view the EOS as an ordered set of steps $A_{i}={\left\{\left(r_{i,m},u_{i,m},\chi_{i,m}\right)\right\}}_{m=1}^{M_{i}}$, where $M_{i}\in N$ is the number of steps, $r_{i,m}\in R$ is a role label from a finite set R (e.g., driver/dispatcher/station staff), $u_{i,m}\in \Sigma^{*}$ is the step action text, and $\chi_{i,m}$ denotes a set of step conditions (e.g., prerequisites or stop conditions). The EOS text $a_{i}$ is the serialization of $A_{i}$ under a fixed schema, so the final supervision dataset is $D_{QA}={\left\{\left(q_{i},a_{i}\right)\right\}}_{i=1}^{N}$.

Retrieval augmentation requires an evidence store. We construct a knowledge base K as the union of a chunked document memory $K_{\mathrm{doc}}={\left\{d_{j}\right\}}_{j=1}^{J}$ with J∈N chunks and an optional maintenance knowledge graph $K_{\mathrm{kg}}$. Formally,

(9) $$K=K_{\mathrm{doc}}\cup K_{\mathrm{kg}}.$$

Online, given a new incident with observation $\left(x^{\left(b\right)},x^{\left(c\right)},x^{\left(o\right)},\pi ,t\right)$, we compute z by (1)–(5), synthesize a query *q* by (6), retrieve an evidence context $C^{★}\left(q\right)\subseteq K$, and generate an EOS $\hat{a}\in \Sigma^{*}$ using a conditional generative model $p_{\theta }\left(\cdot \right)$ with parameters θ:

(10) $$\hat{a}=\arg\max_{a\in \Sigma^{*}}\log p_{\theta }\left(a|q,C^{★}\left(q\right)\right).$$

The rest of the methodology details how θ is learned (Section 3.2) and how $C^{★}\left(q\right)$ is constructed and used under constraints (Section 3.3).

### 3.2. Fine-Tuning of the LLM for EOS Generation

The generative component is adapted to the EOS domain by supervised fine-tuning on $D_{QA}$, while explicitly encouraging structured outputs and evidence-grounded decisions. Let $a_{i}=\left(w_{i,1},\ldots ,w_{i,T_{i}}\right)$ denote the token sequence of the EOS answer for sample *i*, where $T_{i}\in N$ is the sequence length and each token $w_{i,t}\in \Sigma$. Let the packed conditioning context be $c_{i}=\mathrm{Pack}\left(q_{i},C^{★}\left(q_{i}\right)\right)\in \Sigma^{*}$, where Pack(·) is a deterministic formatting operator that concatenates the question and retrieved evidence into a single prompt. The base learning signal follows teacher forcing via the negative log-likelihood (NLL),

(11) $$L_{\mathrm{NLL}}\left(\theta \right)=\frac{1}{N}\sum_{i=1}^{N}\frac{1}{T_{i}}\sum_{t=1}^{T_{i}}-\log p_{\theta }\;\left(w_{i,t}|w_{i,<t},c_{i}\right),$$

where $w_{i,<t}=\left(w_{i,1},\ldots ,w_{i,t-1}\right)$ is the prefix. While (11) aligns surface text, EOS quality also depends on whether the generated plan respects an expected schema and role sequencing. We therefore treat EOS generation as a constrained sequence problem with a latent step structure. Let $G_{\mathrm{sch}}$ be a finite schema grammar that defines admissible EOS structures (e.g., required section headers, step numbering, mandatory role tags, and evidence citation markers). For a candidate EOS *a*, let $\mathrm{viol}(a;G_{\mathrm{sch}})\in N$ count schema violations. Since viol(·) is not directly differentiable, we incorporate it via a sampling-based expected penalty. Let ${\tilde{a}}_{i}∼p_{\theta }\left(\cdot |c_{i}\right)$ be a decoded sample; then

(12) $$L_{\mathrm{sch}}\left(\theta \right)=\frac{1}{N}\sum_{i=1}^{N}E_{{\tilde{a}}_{i}∼p_{\theta }\left(\cdot |c_{i}\right)}\left[\mathrm{viol}({\tilde{a}}_{i};G_{\mathrm{sch}})\right].$$

To couple the generated text with the structured step representation $A_{i}=$${\left\{\left(r_{i,m},u_{i,m},\chi_{i,m}\right)\right\}}_{m=1}^{M_{i}}$, we regularize role emissions. Let $M_{i}\in N$ be the number of steps, and let pos(m,a)∈{1,…,|a|} map step *m* to a token position in serialization, where |a|∈N denotes the token length of *a*. With R the role vocabulary and $r_{i,m}\in R$, we define a role prediction loss

(13) $$L_{\mathrm{role}}\left(\theta \right)=\frac{1}{N}\sum_{i=1}^{N}\frac{1}{M_{i}}\sum_{m=1}^{M_{i}}-\log p_{\theta }\;\left(r_{i,m}|w_{i,<\mathrm{pos}(m,a_{i})},c_{i}\right).$$

Evidence traceability is enforced by training the model to emit citations that point to retrieved chunks. Let $C^{★}\left(q_{i}\right)=\left\{d_{i,1},\ldots ,d_{i,k}\right\}$ be the selected evidence set of size k∈N, and let $g_{i,m,j}\in \left\{0,1\right\}$ indicate whether EOS step *m* should be supported by evidence chunk $d_{i,j}$. The model produces citation logits $s_{i,m,j}\in R^{k}$ for step *m*, where $s_{i,m,j}$ is the logit corresponding to chunk *j*. We use a multi-label logistic loss

(14) $$L_{\mathrm{cite}}\left(\theta \right)=\frac{1}{N}\sum_{i=1}^{N}\frac{1}{M_{i}}\sum_{m=1}^{M_{i}}\sum_{j=1}^{k}\left(\log\left(1+\exp\left(s_{i,m,j}\right)\right)-g_{i,m,j}s_{i,m,j}\right).$$

The overall fine-tuning objective combines likelihood, schema, role, and citation penalties. Let $\lambda_{\mathrm{sch}},\lambda_{\mathrm{role}},\lambda_{\mathrm{cite}}\geq 0$ be scalar weights, and let ${∥\theta ∥}_{2}^{2}$ denote an $l_{2}$ regularizer with coefficient $\lambda_{\mathrm{reg}}\geq 0$. Training minimizes

(15) $$\min_{\theta }\;L\left(\theta \right)=L_{\mathrm{NLL}}\left(\theta \right)+\lambda_{\mathrm{sch}}L_{\mathrm{sch}}\left(\theta \right)+\lambda_{\mathrm{role}}L_{\mathrm{role}}\left(\theta \right)+\lambda_{\mathrm{cite}}L_{\mathrm{cite}}\left(\theta \right)+\lambda_{\mathrm{reg}}{∥\theta ∥}_{2}^{2}.$$

Because domain adaptation may be computationally demanding, we implement parameter-efficient tuning via low-rank adapters. Consider any linear projection in the base model with weight matrix $W_{0}\in R^{d_{\mathrm{out}}\times d_{\mathrm{in}}}$, where $d_{\mathrm{in}}\in N$ and $d_{\mathrm{out}}\in N$ are input and output dimensions. LoRA parameterizes $W=W_{0}+\Delta W$, with ΔW=BA, where $A\in R^{r\times d_{\mathrm{in}}}$, $B\in R^{d_{\mathrm{out}}\times r}$, and r∈N is the adapter rank satisfying $r\ll \min(d_{\mathrm{in}},d_{\mathrm{out}})$. The fine-tuning then updates only A and B while freezing $W_{0}$, which controls overfitting and supports efficient experimentation.

### 3.3. Retrieval-Augmented Generation Modules and Implementation

The RAG subsystem is responsible for selecting evidence that is both relevant to the incident query and compact enough to fit within the model’s context window, while also enabling post hoc verification of EOS steps. We assume that the document memory $K_{\mathrm{doc}}={\left\{d_{j}\right\}}_{j=1}^{J}$ consists of J∈N evidence chunks. Retrieval begins by embedding the query and chunks into a shared vector space. Let $d_{r}\in N$ denote the embedding dimension. A query encoder ${\mathrm{Enc}}_{q}:\Sigma^{*}\to R^{d_{r}}$ maps *q* to $u={\mathrm{Enc}}_{q}\left(q\right)$, and a document encoder ${\mathrm{Enc}}_{d}:\Sigma^{*}\to R^{d_{r}}$ maps $d_{j}$ to $v_{j}={\mathrm{Enc}}_{d}\left(d_{j}\right)$. Dense similarity is measured by cosine score

(16) $$s_{\mathrm{dense}}\left(q,d_{j}\right)=\frac{u^{⊤}v_{j}}{{∥u∥}_{2}{∥v_{j}∥}_{2}},$$

where ${∥\cdot ∥}_{2}$ denotes the Euclidean norm.

Dense retrieval is complemented by lexical matching to better handle operational jargon and component codes. Let $\mathrm{tf}(t,d_{j})\in N$ be the term frequency of token *t* in chunk $d_{j}$, df(t)∈N be the document frequency of token *t* across the *J* chunks, and define inverse document frequency

(17) $$\mathrm{idf}\left(t\right)=\log\frac{J-\mathrm{df}(t)+0.5}{\mathrm{df}(t)+0.5}.$$

Let $|d_{j}|\in N$ denote the token length of chunk $d_{j}$, $\mathrm{avgdl}\in R_{+}$ the average chunk length, and let $k_{1}\geq 0$ and b∈[0,1] be BM25 parameters. The lexical score is then

(18) $$s_{\mathrm{BM}25}\left(q,d_{j}\right)=\sum_{t\in q}\mathrm{idf}\left(t\right)\cdot \frac{\mathrm{tf}\left(t,d_{j}\right)\left(k_{1}+1\right)}{\mathrm{tf}\left(t,d_{j}\right)+k_{1}\left(1-b+b\frac{|d_{j}|}{\mathrm{avgdl}}\right)}.$$

To combine the two signals, we use a convex mixture. Let ${\hat{s}}_{\mathrm{dense}}$ and ${\hat{s}}_{\mathrm{BM}25}$ be min–max normalized scores over j∈{1,…,J}, and let α∈[0,1] be a mixing weight. The hybrid score is

(19) $$s_{\mathrm{hyb}}\left(q,d_{j}\right)=\alpha \;{\hat{s}}_{\mathrm{dense}}\left(q,d_{j}\right)+\left(1-\alpha \right)\;{\hat{s}}_{\mathrm{BM}25}\left(q,d_{j}\right).$$

From (19) we form an initial candidate pool $C_{0}\left(q\right)\subset K_{\mathrm{doc}}$ by taking the top $K_{0}\in N$ chunks, denoted $C_{0}\left(q\right){=}_{d_{j}\in K_{\mathrm{doc}}}s_{\mathrm{hyb}}\left(q,d_{j}\right)$. Because hybrid scoring is still approximate, we rerank candidates with a cross-encoder $\mathrm{Rank}:\Sigma^{*}\times \Sigma^{*}\to R$ that directly models query–chunk relevance, defining $s_{\mathrm{re}}\left(q,d\right)=\mathrm{Rank}\left(q,d\right)$. The final evidence set C(q) is chosen as the top k∈N chunks under $s_{\mathrm{re}}$:

(20) $$C\left(q\right){=}_{d\in C_{0}\left(q\right)}s_{\mathrm{re}}\left(q,d\right).$$

When training dense retrieval components, we align query and document embeddings with a contrastive objective. For each training query $q_{i}$, let $d_{i}^{+}\in K_{\mathrm{doc}}$ be a relevant (positive) chunk and let ${\left\{d_{i,n}^{-}\right\}}_{n=1}^{B-1}\subset K_{\mathrm{doc}}$ be negative chunks sampled within a batch of size B∈N. With temperature $\tau \in R_{+}$, define $u_{i}={\mathrm{Enc}}_{q}\left(q_{i}\right)$, $v_{i}^{+}={\mathrm{Enc}}_{d}\left(d_{i}^{+}\right)$, and $v_{i,n}^{-}={\mathrm{Enc}}_{d}\left(d_{i,n}^{-}\right)$. The InfoNCE loss is

(21) $$L_{\mathrm{ret}}=-\frac{1}{N}\sum_{i=1}^{N}\log\frac{\exp\left(u_{i}^{⊤}v_{i}^{+}/\tau \right)}{\exp\left(u_{i}^{⊤}v_{i}^{+}/\tau \right)+\sum_{n=1}^{B-1}\exp\left(u_{i}^{⊤}v_{i,n}^{-}/\tau \right)}.$$

Evidence selection must also respect a context budget imposed by the LLM. Let $B_{\max}\in N$ be the maximum number of tokens available for evidence in the prompt, and let l(d)∈N denote the token length of chunk *d*. To reduce redundancy among selected chunks, we apply a maximum marginal relevance criterion. Let $\mathrm{sim}\left(d,d^{\prime }\right)\in \left[-1,1\right]$ denote cosine similarity between dense chunk embeddings, and let β∈[0,1] be the relevance–diversity trade-off. Starting from S(q)=∅, we iteratively add the chunk

(22) $$d^{★}=\arg\max_{d\in C(q)\setminus S(q)}\left(\beta \;s_{\mathrm{re}}\left(q,d\right)-\left(1-\beta \right)\max_{d^{\prime }\in S\left(q\right)}\mathrm{sim}\left(d,d^{\prime }\right)\right),$$

subject to the budget constraint $\sum_{d\in S(q)}l\left(d\right)\leq B_{\max}$. The final packed context is $C^{★}\left(q\right)=\mathrm{Pack}\left(S\left(q\right)\right)$, where Pack(·) formats the selected evidence chunks into a deterministic prompt segment.

If a maintenance knowledge graph is available, it can be integrated as an additional retrieval channel. Let $K_{\mathrm{kg}}=\left(V,E,R\right)$ be a directed labeled graph with nodes V, edges E⊆V×R×V, and relations R. Let $d_{g}\in N$ be the graph embedding dimension, and let $e_{v}\in R^{d_{g}}$ and $e_{r}\in R^{d_{g}}$ denote embeddings of node v∈V and relation r∈R. Using a translational scoring function for triple (h,r,t)∈E,

(23) $$\mathrm{score}\left(h,r,t\right)=∥e_{h}+e_{r}-e_{t}{∥}_{2}^{2},$$

with margin $\gamma \in R_{+}$ and negative triples $E^{-}$, the embedding can be learned by

(24) $$L_{\mathrm{kg}}=\sum_{(h,r,t)\in E}\sum_{\left(h^{\prime },r,t^{\prime }\right)\in E^{-}}\max\left(0,\gamma +\mathrm{score}\left(h,r,t\right)-\mathrm{score}\left(h^{\prime },r,t^{\prime }\right)\right).$$

At inference, entity linking maps *q* to a seed distribution $p\in R^{\left|V\right|}$, where $p_{v}>0$ for seeded nodes and $p_{v}=0$ otherwise, and $\sum_{v}p_{v}=1$. Let $A\in R^{\left|V|\times |V\right|}$ be a column-stochastic adjacency matrix and let λ∈(0,1) be a restart probability. Personalized propagation yields relevance vector $r\in R^{\left|V\right|}$ via the fixed point

(25) $$r=\left(1-\lambda \right)p+\lambda A^{⊤}r.$$

The graph-derived evidence can be serialized and appended to $C^{★}\left(q\right)$ as structured hints, yielding a combined context still denoted $C^{★}\left(q\right)$ for notational simplicity.

Finally, to reflect the safety-critical nature of EOS generation, we incorporate compliance as a constrained decoding and verification step. Let ${\left\{\kappa_{m}\right\}}_{m=1}^{M_{\kappa }}$ be a set of $M_{\kappa }\in N$ compliance constraints, and let the satisfaction predicate $a⊧\kappa_{m}$ indicate that EOS *a* satisfies constraint $\kappa_{m}$. Define the violation count

(26) $$V\left(a\right)=\sum_{m=1}^{M_{\kappa }}\left[a⊭\kappa_{m}\right]\in \left\{0,1,\ldots ,M_{\kappa }\right\}.$$

With penalty coefficient $\mu \in R_{+}$, compliance is enforced at inference time by reranking beam search candidates rather than by directly optimizing over $\Sigma^{*}$. Let $A_{\mathrm{beam}}\left(q\right)$ denote the beam candidate set. We then select the final output as

(27) $$\hat{a}=\arg\max_{a\in A_{\mathrm{beam}}\left(q\right)}\left(\log p_{\theta }\left(a|q,C^{★}\left(q\right)\right)-\mu \;V\left(a\right)\right).$$

To quantify evidence faithfulness at the step level, let $\mathrm{Ent}:\Sigma^{*}\times \Sigma^{*}\to \left[0,1\right]$ be an entailment scorer that measures whether a step text is supported by a chunk. For step $u_{m}$ extracted from $\hat{a}$, the maximal support score is

(28) $$\mathrm{supp}\left(u_{m}\right)=\max_{d\in S(q)}\mathrm{Ent}\left(u_{m},d\right),$$

and a threshold ρ∈[0,1] can be used to flag unsupported steps for regeneration or forced citation augmentation. In combination, (16)–(28) formalize a RAG pipeline that is simultaneously relevance-driven, budget-aware, and compliance-sensitive, which is necessary for reliable EOS generation in operational contexts.

### 3.4. Evaluation Protocol and Metrics

Retrieval configuration. We implement hybrid retrieval following (16)–(22). Dense embeddings are computed by a sentence-level encoder for both queries and chunks, and cosine similarity yields $s_{\mathrm{dense}}$. Dense embeddings are computed using Qwen3-Embedding-8B, which is used to encode both queries and document chunks into a shared vector space. Lexical retrieval uses BM25 with standard hyperparameters ($k_{1}$ and *b*), producing $s_{\mathrm{BM}25}$. The final hybrid score uses (19) with a mixing weight α, and the initial candidate pool size is set to $K_{0}$. We then rerank candidates using a cross-encoder relevance model (20), and select the top-*k* chunks under a fixed evidence budget $B_{\max}$ in tokens. Redundancy control uses maximum marginal relevance (22) with diversity coefficient β. Unless otherwise stated, the retrieval setting is fixed to the best-performing configuration reported in Table 6 (Hybrid + rerank), and we retrieve top-k=10 chunks for each query.

Prompt packing and output schema. The packed prompt $c_{i}=\mathrm{Pack}\left(q_{i},C^{★}\left(q_{i}\right)\right)$ follows a deterministic structure with three parts: (i) an instruction specifying the required EOS schema and role tags, (ii) the structured incident context rendered from mixed-type features (time period, line/station, train ID, door index, fault taxonomy path, and impact indicators), and (iii) retrieved evidence chunks with stable chunk identifiers. The EOS answer is constrained to a fixed serialization schema: phases/steps are numbered; each step begins with a role tag in braces (e.g., {Driver}, {Maint. Staff}); and each actionable step must attach at least one citation marker pointing to retrieved chunk identifiers when RAG is enabled.

Evaluation metrics. We evaluate generation quality using four metrics. SchemaPass is the fraction of generated answers with zero schema violations under $G_{\mathrm{sch}}$. RoleAcc measures step-level correctness of emitted roles after parsing the EOS into (role, action, condition) tuples; we report accuracy (or macro-F1 if roles are imbalanced). CiteCov is the fraction of steps that contain at least one valid citation marker pointing to retrieved evidence chunk identifiers. UsableAns is the fraction of outputs that simultaneously satisfy schema validity, role correctness requirements, and (when applicable) citation requirements, and are judged actionable by human raters using a predefined rubric. Retrieval performance is evaluated with Recall@5 (relevance), FirstHit/B (compactness), and Coverage@*B* (verifiability). Here, FirstHit/B is a task-specific compactness metric defined as the normalized position of the first relevant evidence chunk within a fixed evidence budget *B*, i.e., $\mathrm{FirstHit}/B=p_{\mathrm{first}}/B$, where $p_{\mathrm{first}}$ is the rank position of the first relevant retrieved chunk. Therefore, smaller values are better, because they indicate that the first useful supporting evidence appears earlier in the ranked list under the same budget. Coverage@*B* is defined as the fraction of reference EOS-required key operational items (e.g., actions, constraints, or supporting clauses) that are covered by the retrieved evidence within the same budget *B*; thus, larger values are better, because they indicate more complete evidence support for downstream generation and verification. All reported results are averaged over the test set, and ablations differ only in the specified components (RAG and/or structured fine-tuning losses).

## 4. Data and Experimental Settings

### 4.1. Data

**Figure 2:**
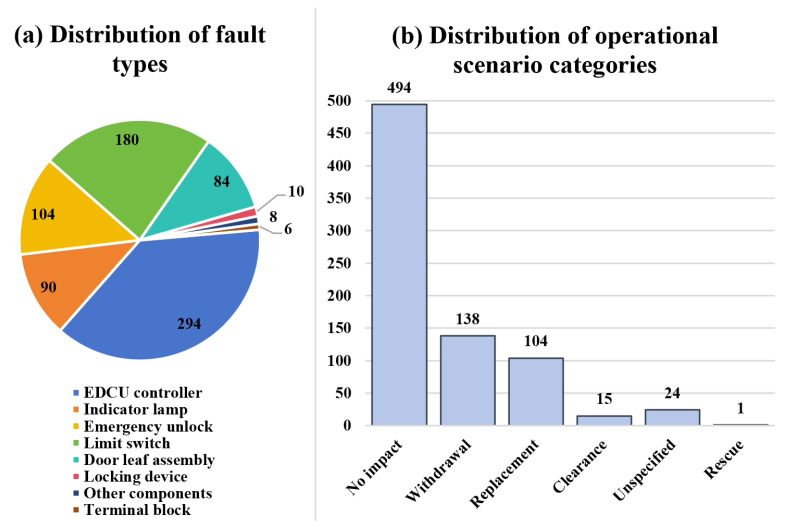
Overview of the 776 train-door failure cases used in this study: (**a**) distribution of major fault types; (**b**) distribution of operational scenario categories.

The empirical analysis is based on multi-source heterogeneous operational data collected from the Shanghai urban rail transit (URT) system over a six-year period spanning 1 January 2019 to 31 December 2024. The raw data cover three complementary views of door-related incidents: (i) structured rolling-stock maintenance work orders that record fault identifiers, timestamps, train identifiers, and hierarchical component classifications; (ii) structured operations management and dispatching records that describe operational impacts such as delay propagation, short-turn/turn-back adjustments, and passenger-flow abnormality handling; and (iii) unstructured textual narratives, including maintenance handling logs and operations incident reports, which preserve fine-grained action sequences and contextual details that are typically absent from structured fields. After cleaning and feature reconstruction, the study focuses on 776 passenger-door subsystem events and their associated operational impact features. Figure 2 further summarizes the composition of these cases from two perspectives: the distribution of major fault types and the distribution of operational scenario categories. As shown in Figure 2a, the dataset is dominated by several recurrent component-level fault types, such as EDCU controller, limit switch, emergency unlock, and indicator lamp failures, while Figure 2b shows that most incidents are low-impact cases with no service impact, followed by withdrawal and replacement scenarios. This distribution indicates that the dataset covers both high-frequency technical fault modes and multiple operational consequence settings, thereby supporting downstream construction of question–answer supervision for large language model (LLM) training and retrieval-augmented generation (RAG).

Table 1 summarizes the schema of the maintenance work-order data for passenger-door failures. Each record includes a fault code (used as the primary event identifier), occurrence time, train number, door type, and a multi-level fault taxonomy (level-1/2/3 categories) with a specific failed component. Table 2 reports the operational impact statistics aligned to the same fault code, including binary context indicators (e.g., whether the station environment is adjacent to an escalator) and count variables that quantify delay occurrences under multiple delay-duration bins as well as operational interventions such as turn-back and passenger clearance. Table 3 provides examples of the unstructured text logs, where maintenance narratives and dispatching narratives jointly describe “symptom–action–outcome” trajectories that are essential for extracting ground-truth emergency operation schemes (EOSs).

Data preprocessing consists of three steps. First, door-related event filtering is applied to retain only incidents directly associated with the passenger-door subsystem. This filtering is implemented by restricting the system-function and sub-function labels to door-specific categories (e.g., mechanical components, electrical components, and auxiliary components of passenger doors) and discarding records that do not correspond to door failures. Second, duplicate removal and missing-value treatment are performed at the event level. Duplicate work orders are removed, while missing entries in categorical fields (e.g., a placeholder such as “–” in level-2 classification) are imputed using the within-group mode under the same higher-level category, which preserves the hierarchical consistency of the taxonomy. After these operations, 776 valid door-failure events remain for analysis. Third, temporal alignment is conducted by matching the fault occurrence time to the corresponding train timetable and dispatch logs, ensuring the chronological consistency between a door-failure event and its associated delay and operational-adjustment records.

A notable characteristic of this dataset is that the non-text features are mixed-type, and therefore cannot be treated as raw text inputs without explicit encoding. Specifically, as in Table 4, the operational impact labels are ordinal (reflecting increasing delay severity), several contextual indicators are binary, some operational statistics are continuous or count-valued, and the fault location is inherently hierarchical (tree-structured) through multi-level component taxonomy. To make these signals usable for LLM-driven EOS generation, we encode the hierarchical fault attributes as a tree/path representation, while binary/ordinal/continuous variables are converted into a standardized structured representation that can later be rendered into natural-language. These variables, together with the textual narratives, are then transformed into question–answer supervision pairs: the question describes the incident context (fault symptoms, location in the taxonomy, time/space context, and impact indicators), whereas the answer is the canonicalized EOS extracted from the maintenance and dispatch narratives. This design explicitly preserves both the structured operational semantics and the textual action traces, which are required for robust EOS generation and subsequent evidence-grounded verification.

To quantify the operational-delay impact of door failures, the dependent variable is defined as an ordinal severity label with four ordered categories, constructed from observed delay duration and major operational interventions (Table 4). The independent variables include temporal context (peak vs. off-peak), station type (hub vs. non-hub), station environmental risk proxy (adjacency to escalators), and door-fault component features represented by a three-level tree-structured encoding. Binary covariates are one-hot encoded, continuous covariates are standardized, and hierarchical covariates are represented by taxonomy-path indicators. For unobserved confounders (e.g., equipment aging, ambient temperature/humidity), the modeling stage further adopts regularization and propensity-score-based adjustments to mitigate bias introduced by missing covariates.

### 4.2. Experiment Settings

This section reports the experimental configuration for retrieval, fine-tuning, and evaluation. All experiments are conducted at the incident (fault-code) level, ensuring that all structured fields and corresponding text logs of a single event are always kept in the same split.

Data split and protocols. The cleaned dataset contains N=776 validated passenger-door failure events. We adopt a stratified split by delay-severity label to mitigate imbalance: 60% for training, 20% for validation, and 20% for testing. All ablations (B0–B4) use identical splits and identical retrieval settings (for methods with RAG) to ensure fair comparison.

Knowledge base construction for retrieval. The document memory $K_{\mathrm{doc}}$ is built from: (i) maintenance regulations and operational manuals (including the “Urban Rail Vehicle Maintenance Regulations”), employee training and operating manuals, and internal summary reports; (ii) standard operating procedures for door isolation/cut-out; and (iii) historical canonicalized EOS/MOS cases derived from maintenance and dispatch logs. The historical case memory used for retrieval was constructed from 776 train-door failure incidents collected from the Shanghai Metro between 2019 and 2024. Before the materials were accessed by the researchers, sensitive operational identifiers had already been anonymized by the data provider, for example by replacing actual train numbers and equipment identifiers with secondary coded representations. Duplicate records were removed during preprocessing. Document cleaning further removed repeated headers/footers and scanning noise, and all files were normalized to a unified UTF-8 encoding and formatting standard. Clause and section numbering were preserved whenever available to maintain traceability to the original rules and manuals. For retrieval indexing, the cleaned documents were segmented using dynamic chunking, in which narrative paragraphs and tabular content were processed separately, with an overlap ratio of 10% between adjacent chunks to reduce information fragmentation across chunk boundaries. This process yielded approximately 1800 retrievable chunks. Each chunk retains only its original document source as metadata. To further support reproducibility and help readers understand the data organization used in the framework, we additionally provide an anonymized sample data table in the revised manuscript/Supplementary Materials. In all RAG-enabled settings of this study, the knowledge base further includes a maintenance knowledge graph channel $K_{\mathrm{kg}}$, which is constructed from a component taxonomy and fault–action relations extracted from structured work orders and canonicalized handling records. Rather than serving as an independently toggled module, $K_{\mathrm{kg}}$ is treated as a fixed component of the RAG subsystem and is used to complement document retrieval with structured fault–component–action relations. The graph-derived evidence is serialized as structured triples and appended to the retrieved document context as structured hints before EOS generation.

LLM backbone and fine-tuning. B0–B1 use a fixed commercial LLM API Qwen3.5-Flash as a black-box generator without parameter updates. B2–B4 use an open-source causal LLM backbone, namely Qwen3.5-9B, and adapt it to the EOS generation task through parameter-efficient supervised fine-tuning. Specifically, LoRA adapters are inserted into the linear projections of each transformer block, including the self-attention projections and the feed-forward projections. The LoRA rank is set to r=8, the scaling factor is $\alpha_{\mathrm{LoRA}}=32$, and only adapter parameters are updated while the backbone weights remain frozen. We train with teacher forcing using the objective in (15). The structured losses are enabled as follows: B2 uses $L_{\mathrm{NLL}}$ only; B3 adds $L_{\mathrm{sch}}$ and $L_{\mathrm{role}}$; B4 additionally adds $L_{\mathrm{cite}}$. Therefore, the adaptation is not merely format imitation, but a structure-aware domain fine-tuning process that explicitly optimizes schema compliance, role assignment consistency, and citation grounding for safety-critical EOS generation. Hyperparameters $\left(\lambda_{\mathrm{sch}},\lambda_{\mathrm{role}},\lambda_{\mathrm{cite}}\right)$ are selected on the validation set by maximizing the overall usability metric (UsableAns). Training uses AdamW with a learning rate of $1\;\times \;10^{-4}$, a batch size of 16, and 5 epochs.

Compliance-aware decoding and verification. At inference, decoding uses beam search or constrained sampling with a compliance penalty as in (27). Compliance constraints $\left\{\kappa_{m}\right\}$ include: mandatory schema headers/step numbering, mandatory role tags, and citation requirements for RAG-enabled settings. We further compute step-level evidence support (28) using an entailment scorer between each generated step and the retrieved evidence chunks. Steps with support below a threshold ρ are flagged as unsupported and contribute to error analysis; optionally, unsupported steps trigger regeneration with strengthened citation prompts (not enabled in the main results unless explicitly noted).

For further reproducibility, Table 5 summarizes the main hyperparameters used in retrieval, fine-tuning, and decoding.

Human evaluation protocol. Human evaluation of UsableAns was conducted on the test set (approximately 150 cases) through a paid expert questionnaire. A total of 10 domain experts participated in the evaluation, including dispatchers and frontline operational/maintenance personnel. Due to confidentiality and privacy regulations, the detailed identities of the participants cannot be disclosed in the manuscript. To control annotation burden associated with reading full EOS outputs, each expert was assigned approximately 15 cases for assessment, and each case was evaluated once by a single expert. The experts judged whether a generated answer was operationally usable according to a predefined rubric covering schema validity, role assignment correctness, step executability, and citation compliance when applicable. Specifically, an output was marked as usable only when it followed the required EOS structure, assigned responsibilities to appropriate roles, contained operationally executable and non-contradictory actions, and satisfied citation requirements in RAG-enabled settings. Because each case received only one expert judgment, inter-rater agreement statistics were not available in the present study. To improve statistical transparency, we therefore report confidence intervals for UsableAns in the revised manuscript.

## 5. Results

This section reports the empirical performance of the proposed RAG-enhanced EOS generation framework, focusing on three questions: (i) whether the retrieval module can reliably return relevant and compact evidence to support downstream generation; (ii) whether supervised fine-tuning with structured objectives improves the executability and standardization of generated emergency operation schemes (EOS); and (iii) where and why the system fails under operationally difficult cases. Then, we present qualitative case studies to demonstrate how retrieved clauses are packed into prompts and how the model produces step-wise, role-tagged, and cite-grounded outputs (Tables 8 and 9). Overall, the results indicate that stronger retrieval (hybrid + rerank) and structured fine-tuning objectives jointly improve not only text-level quality but also operational usability, by reducing low-level formatting errors and increasing evidence-grounded steps in safety-critical responses.

### 5.1. Retrieval Performance

In safety-critical EOS generation, retrieval quality directly determines whether the model can produce responses that are not only fluent but also actionable and verifiable. Table 6 reports the evaluation of different retrieval strategies. Dense-only retrieval achieves a moderate Recall@5 of 0.63, indicating that semantic matching can recover relevant chunks for many incidents. However, its compactness is relatively weak (FirstHit/B=0.71), which implies that the first relevant evidence often appears late in the ranked list. In practice, this behavior is unfavorable for EOS generation because delayed exposure to the correct handling rule increases the likelihood that early decoding commits to incomplete or non-compliant steps. BM25-only retrieval performs worse on relevance (0.55) and verifiability (0.54), suggesting that lexical matching alone is sensitive to variations in operational wording, abbreviations, and component codes, and therefore misses relevant precedents even when the underlying fault is similar.

The hybrid strategy provides a clear improvement by combining dense semantics with lexical cues: Recall@5 increases to 0.71 and Coverage@*B* rises to 0.66, while FirstHit/B decreases to 0.55. These results indicate that hybrid retrieval better satisfies the evidence needs of high-quality EOS generation: it not only finds more relevant clauses but also places the first relevant clause earlier, leaving more context budget for complementary supporting evidence. Importantly, adding cross-encoder reranking further strengthens this advantage. Hybrid + rerank yields the best overall retrieval performance, with the highest Recall@5 (0.78) and Coverage@*B* (0.71), together with the lowest FirstHit/B (0.46). This means that, relative to other baselines, our evidence selection is more likely to (a) include the correct operational guidance within the top-5 retrieved chunks, (b) surface it early enough to be effectively used during generation under a fixed budget, and (c) provide broader coverage of key actions and entities required by the reference EOS.

Overall, the results support the design choice of a hybrid retriever complemented by reranking. Compared with dense-only and BM25-only baselines, our retrieval module better aligns with the practical requirements of safety-critical EOS generation, where compact, relevant, and coverage-rich evidence is necessary to produce standardized steps and to enable downstream citation-based verification.

### 5.2. EOS Generation Quality

**Figure 3:**
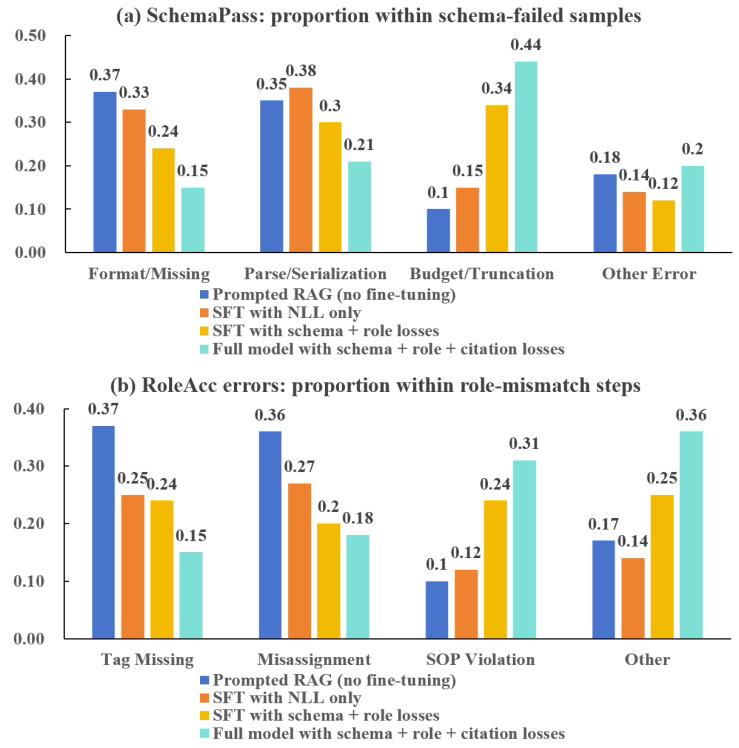
Error analysis of the proposed framework. (**a**) The proportion of failure types within SchemaPass-failed samples. (**b**) The proportion of error types within role-mismatch steps.

Table 7 reports EOS generation quality under progressively stronger settings, and Figure 3 further explains the dominant failure sources. High-quality EOSs in this study are defined by three operational requirements: the output must follow a fixed schema, assign correct responsibilities to roles at the step level, and remain evidence-grounded through explicit citations. Under these criteria, the pure LLM API baseline (B0) performs poorly, with low SchemaPass (0.28), low RoleAcc (0.35), and a very limited fraction of fully usable answers (UsableAns = 0.15), indicating that generic generation without domain adaptation cannot consistently produce standardized and actionable procedures.

Introducing retrieval augmentation without fine-tuning (B1) improves all applicable metrics, raising SchemaPass from 0.28 to 0.47 and RoleAcc from 0.35 to 0.52, while enabling citation coverage (CiteCov = 0.40). However, the overall usability remains limited (UsableAns = 0.26), showing that prompting with retrieved evidence alone is insufficient to guarantee structured and role-consistent outputs. In contrast, supervised fine-tuning on domain question–answer pairs (B2) yields a larger improvement in procedural correctness (SchemaPass = 0.58; RoleAcc = 0.69) and usability (UsableAns = 0.41), confirming that domain adaptation is necessary for stable EOS formatting and role emission.

The main performance gains arise when structured objectives are introduced. Adding schema and role losses (B3) leads to a substantial jump in SchemaPass (0.81) and RoleAcc (0.83), and increases UsableAns to 0.66. This indicates that explicitly optimizing for schema validity and role correctness is effective in reducing low-level procedural errors that directly prevent operational use. The full model (B4) further improves all dimensions, achieving the best overall quality (SchemaPass = 0.88; RoleAcc = 0.91; CiteCov = 0.73; UsableAns = 0.83). Compared with the strongest baseline without citation loss (B3), B4 improves CiteCov by 0.17 and UsableAns by 0.17, showing that citation-aware fine-tuning strengthens evidence traceability and increases the fraction of outputs that can be directly adopted by practitioners.

Figure 3 shows that our full setting (B4) mainly reduces low-level, factual, and structural errors that directly make an EOS unusable. For schema failures (Figure 3a), B1–B2 are dominated by Format/Missing and Parse/Serialization (e.g., B1: 0.37/0.35; B2: 0.33/0.38), reflecting missing required fields and inconsistent step formatting. Under B4, these two error types drop to 0.15 and 0.21, indicating that schema- and role-aware training effectively prevents common preventable mistakes and produces more complete, standardized outputs. After these errors are controlled, the remaining schema failures are largely Budget/Truncation (0.44), suggesting that the main residual limitation is context length rather than formatting correctness.

A similar trend appears for role mismatches (Figure 3b). Tag Missing and Misassignment decrease from 0.37 to 0.15 and from 0.36 to 0.18, showing that our method reduces basic responsibility-label errors. As a result, the remaining errors are relatively more concentrated in SOP Violation (0.10 → 0.31) and Other (0.17 → 0.36). This does not mean worse performance; it means low-level errors are removed, so the residual cases are mainly higher-level judgment errors that require stronger compliance checking.

Overall, Table 7 and Figure 3 together show that the proposed full framework (B4) outperforms all baselines by jointly improving standardization (schema compliance), role correctness, and evidence grounding, which directly translates into a higher proportion of operationally usable EOS outputs.

### 5.3. Samples of EOS Generation

**Figure 4:**
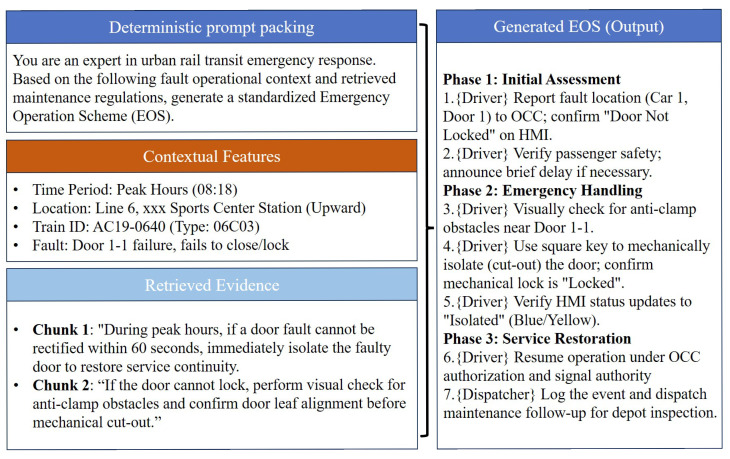
Example of structured input packing and EOS generation in the proposed RAG-enhanced system.

Figure 4 presents a compact illustration of the packed prompt-to-EOS pipeline, showing how deterministic prompt construction, structured contextual features, and retrieved evidence are combined to produce a phase-wise and citation-grounded EOS output. Table 8 and Table 9 illustrate why the proposed framework already meets key requirements for practical deployment in URT emergency operations.

First, the input side of the framework is operationally realistic and structurally explicit. As shown in Figure 4, the packed prompt is composed of three parts: a deterministic task instruction, a set of routinely available contextual features, and compact retrieved evidence chunks. The contextual features rely on standard operational fields such as time period, location, train identifier, and fault symptom/diagnosis, and therefore do not require additional sensors or complex manual feature engineering. The retrieved evidence is represented as compact regulation- or case-derived chunks, which can be directly appended to the structured input before generation. This design lowers the barrier to integration with OCC/dispatch and maintenance information systems, while keeping the input representation consistent with how incidents are reported and handled in practice (Table 8 and Table 9).

Second, the outputs are directly executable because they are organized into phases and serialized as ordered steps with explicit role labels. In the on-site scenario, the generated EOS separates initial assessment, emergency handling, and service restoration, and assigns each step to the appropriate operator (e.g., driver actions for isolation and status confirmation) (Table 8). In the depot scenario, the generated MOS follows a typical maintenance workflow from diagnosis and preparation to component replacement and verification/closure, and distinguishes responsibilities between maintenance staff and quality inspection where needed (Table 9). Such structure is essential for reducing ambiguity under time pressure and supporting human–machine collaboration. The on-site case emphasizes rapid decision-making and service recovery within operational constraints, whereas the depot case emphasizes correctness of technical procedures and closure quality.

Overall, these samples indicate that the proposed system can produce standardized, role-consistent, and evidence-traceable schemes under realistic inputs, which constitutes the basic practical value of deploying an LLM+RAG assistant for URT door-fault emergency operations.

## 6. Discussion

This study shows that EOS generation quality in safety-critical URT operations is primarily constrained by evidence usability and output executability rather than surface-level fluency. On the retrieval side, the Hybrid + rerank setting achieves the strongest evidence selection performance (Recall@5 = 0.78; Coverage@*B* = 0.71) while also surfacing the first relevant clause earlier under a fixed budget (FirstHit/B=0.46) (Table 6). On the generation side, structured fine-tuning objectives yield the main improvement in operational usability: compared with unstructured SFT (B2), adding schema and role losses (B3) increases SchemaPass from 0.58 to 0.81 and raises UsableAns from 0.41 to 0.66, indicating that many failures in earlier settings are “low-level” and preventable. The full setting (B4) further improves UsableAns to 0.83 and increases CiteCov to 0.73 (Table 7), which is consistent with the requirement that usable EOS must be both executable and verifiable. Error analysis (Figure 3) clarifies that the framework effectively suppresses low-level factual and structural errors.

The observed gains are consistent with a “retrieval-first, structure-second” dependency in EOS generation. First, Hybrid retrieval improves relevance and coverage by combining dense semantics with lexical matching, which is important in URT logs where component codes, abbreviations, and line-specific terminology can be poorly handled by either dense-only or BM25-only retrieval. Cross-encoder reranking then refines the candidate pool by modeling query–chunk relevance more directly, which explains why Hybrid + rerank not only improves Recall@5 but also reduces FirstHit/B (Table 6). This earlier exposure to the correct clause is practically important: under a fixed context window, an EOS is more likely to remain compliant when the model encounters the key operational rule before committing to early steps.

Structured objectives reduce missing mandatory fields and serialization errors, while the role loss reduces responsibility misassignment at the step level, which directly aligns with executability requirements (Table 7). Figure 3 supports this interpretation: categories associated with preventable formatting and labeling errors shrink substantially under B4, indicating that these errors are not an unavoidable consequence of generative modeling but can be reduced by explicitly training toward the required output constraints.

From an applied perspective, the main value of the proposed framework is that it produces EOS outputs that are closer to being directly deployable within OCC/dispatch and maintenance workflows. First, the combination of evidence usability (Hybrid and rerank) and structured generation makes responses more executable under time pressure, because steps are serialized, role-assigned, and less likely to contain missing fields or ambiguous formatting. Second, explicit citation attachment supports verifiability and accountability: operators can quickly locate the supporting clause in the retrieved manuals, which is necessary for safety audits, post-incident review, and standard-compliance assurance. Overall, these findings suggest that improving “evidence usability and executability constraints” is a more effective route to production-grade EOS assistance than optimizing generic text similarity metrics, because the primary adoption barrier in URT emergency operations is whether an output can be executed and verified, not whether it is linguistically similar to a reference narrative.

It is important to clarify the intended deployment boundary of the framework. The proposed system is designed as a human-in-the-loop decision-support assistant for trained operational personnel, not as an automated actuator for direct door control. In current practice, door-fault handling is executed by personnel working under predefined EOSs, SOPs, operating manuals, and dispatch/maintenance workflows; the value of the present framework is to make these responses faster to access, more role-consistent, and more traceable to supporting clauses. In this sense, the system supports operational judgment and coordination under time pressure, while final responsibility for execution remains with the authorized staff.

## 7. Conclusions

URT train-door failures are safety-critical and operationally disruptive, and they require emergency operation schemes that are not only fluent but also executable, role-clear, and verifiable against regulations. To address this need, this study proposed a retrieval-augmented large language model (LLM) framework for generating standardized operation schemes. The framework (i) normalizes heterogeneous incident evidence into a structured incident representation, (ii) retrieves compact and relevant clauses from regulations and historical cases using a hybrid retriever with reranking, and (iii) generates step-wise schemes under explicit constraints on schema, role assignment, and evidence citation.

Main findings. On the retrieval side, the hybrid retriever with cross-encoder reranking provides the most usable evidence for generation, improving relevance and coverage under a fixed context budget (e.g., Recall@5 and Coverage@*B* in Table 6). On the generation side, supervised fine-tuning is necessary for stable operational outputs: compared with prompting-only baselines, adding structured objectives for schema and role correctness substantially increases the fraction of executable answers, and further adding citation-aware training improves evidence traceability. Error analysis indicates that the full setting mainly suppresses low-level failures that prevent deployment (missing fields, inconsistent serialization, and role-tag errors), after which the dominant remaining failure modes shift to context-budget truncation and higher-level SOP judgment inconsistencies in subtle conditions.

Contributions. This work makes four contributions. First, it presents an end-to-end, deployment-oriented pipeline for EOS (and MOS as an extension) generation, integrating structured incident representation, hybrid retrieval with reranking, constrained decoding, and evidence-grounded verification. Second, it proposes structured fine-tuning objectives that directly target operational executability by jointly optimizing schema validity, role assignment, and citation grounding, rather than relying on generic language modeling loss alone. Third, it establishes a usability-centered evaluation protocol, including SchemaPass, RoleAcc, CiteCov, and the overall UsableAns metric, together with an interpretable error taxonomy that explains how and why outputs fail under different settings. Fourth, it validates the proposed framework on a real-world multi-source dataset of passenger-door incidents from Shanghai URT (2019–2024) and provides case studies showing that the generated schemes can be organized into phases, assigned to proper roles, and linked to supporting evidence, which collectively meets the minimum requirements for decision-support use in safety-critical operations.

The current framework is constrained by (i) context-length limits that can truncate mandatory sections under long evidence, (ii) incomplete or imperfect evidence coverage when the knowledge base lacks up-to-date clauses or rare corner cases, and (iii) sensitivity to regulation versioning and terminology shifts. In addition, confidentiality constraints on real operational logs may limit public release of data and full reproducibility. Future work will follow three practical directions. First, to reduce truncation, we will develop budget-aware long-form generation via multi-stage decoding (generating a mandatory skeleton first), evidence compression and prioritization, and stricter constraints to guarantee required fields. Second, to mitigate SOP-level inconsistencies, we will strengthen compliance verification through rule-based checkers, cross-step and cross-role consistency validation, and multi-pass self-checking to detect and revise unsafe or contradictory actions before finalization. Third, to support broader deployment, we will study cross-line/cross-city transfer with configurable role and taxonomy mappings, continuous knowledge-base updates with explicit regulation version control, and online monitoring to track emerging failure modes and trigger targeted retraining or retrieval updates.

## Figures and Tables

**Table 1:** Fault data in urban rail transit train door systems.

Fault Code	Occurrence Time	Train No.	Sub-Function	Base Function	Door Type	Level-1 Category	Level-2 Category	Level-3 Category	Specific Failed Component
1	9 January 2019, 08:30:15	AC01-117	Passenger-door mechanical	Door limit switch	Pneumatic built-in door	Electrical	Mechanism subcomponent damage	Limit switch	Door close limit switch
2	15 January 2019, 09:23:17	AC06-145	Passenger-door electrical	Door control unit and circuits	Electric external door	Electrical	Mechanism subcomponent damage	–	Door controller (EDCU)
3	18 January 2019, 17:30:25	AC01-122	Passenger-door electrical	Auxiliary component	Pneumatic built-in door	Electrical	Mechanism subcomponent damage	Indicator lamp	Indicator lamp
*…*									
774	5 December 2024, 19:16:45	DC01-107	Passenger-door mechanical	EDCU electronic controller	Electric external door	Electrical	Mechanism subcomponent damage	Door controller EDCU	Door controller (EDCU)
775	19 December 2024, 17:20:57	AC06-147	Passenger-door mechanical	Locking device	Pneumatic built-in door	Mechanical	Mechanism subcomponent damage	Locking device	Door hook
776	27 December 2024, 20:42:38	AC06-142	Passenger-door electrical	Door control unit and circuits	Electric external door	Electrical	Mechanism subcomponent damage	Door controller EDCU	Door controller (EDCU)

**Table 2:** Statistics of the operational impact of urban rail transit train door faults on operations.

Fault Code	Adjacent to Escalator	Terminal Delay ≤2 min (Count)	Cancelled Trains (Count)	Extra Trains (Count)	Delay 5–15 min (Count)	Delay 15–30 min (Count)	Passenger Clearance Fault (Count)	Passenger Clearance Adjustment (Count)	Total Passenger Clearance (Count)
1	No	0	1	0	0	0	0	0	0
2	No	3	0	0	0	0	0	0	0
3	Yes	2	0	0	0	0	0	0	0
*…*									
774	Yes	3	1	2	0	0	1	0	1
775	No	4	2	2	0	0	1	0	1
776	Yes	7	3	3	2	2	0	0	0

**Table 3:** Examples of maintenance handling logs and operations incident logs (text data).

Vehicle Maintenance Log	Operations Incident Log
Work order: Line 6 (temporary), 1 January 2019, 08:18. At Yuanshen Sports Center Station (up direction), train 0640#, operating cab TC1, passenger door 1-1 failure; the door has been isolated (cut out).	08:18 The driver reported a passenger-door 1-1 failure in cab TC1 of train 0640# on the up direction. The traffic controller instructed the driver to conduct on-site isolation (cut-out) and notified vehicle dispatch.
	08:20 The driver confirmed the door had been cut out; the traffic controller instructed the driver to restore the correct driving mode and depart under signal authority.
Work order: Line 1 (temporary), 3 January 2019, 13:50. At Shanghai Railway Station (down direction), train 0135#, passenger-door failure at car 8, door 5.	13:50 Shanghai Railway Station (down), service 15396, train 0135#. The driver reported a passenger-door failure at the last door of the last car; repeated open/close attempts did not clear the fault. The OCC traffic controller instructed the driver to carry a handheld radio and conduct on-site handling, and notified vehicle dispatch.
	13:55 After on-site handling, the driver isolated (cut out) the left-side car 8 door 5 and the train resumed operation.
	4 January 2019, 03:05 After the train returned to depot, CCTV review indicated foreign-object intrusion at door sill of door 2 on car 01601; the driver removed the object and isolated the door. Depot inspection found no abnormalities in the door cylinder, uncoupling small cylinder, limit switches, related components, or critical dimensions; repeated door cycling tests were normal.
Work order: Line 5 (temporary), 7 January 2019, 14:23. At Dongchuan Road Station (up direction), train 0508#. Car 3 door 3 displayed a yellow door status (intermittent fault indication).	14:23 Minhang Development Zone (up), service 0581MH, train 0508#. Driver (Lu Bin-feng) reported that the DDU in cab TC1 indicated a yellow door status for car 3 door 3; all door interlock (closed/locked) indicators were illuminated.
	14:28 Maintenance staff reported that the fault cleared after restarting subsystem S4 and the train met the operational conditions.
	14:33 The driver reported again that the DDU indicated a yellow door status for car 3 door 3; door interlock indicators were not illuminated. The traffic controller instructed isolation (cut-out) of the faulty door while continuing operation and notified vehicle dispatch.
	14:35 The driver confirmed the faulty door had been cut out; door interlock indicators returned to normal and the train resumed movement. The traffic controller instructed downstream stations to implement relevant passenger-service measures.
	17:16 Maintenance staff reported that the door DCU was replaced and the fault was rectified; the train met the operational conditions, with follow-up depot inspection required (not yet fully closed out).
	02:30 Vehicle dispatch reported that the car 3 door 3 yellow indication was confirmed as a failed DCU (MP2 A2). The MP2 A2 passenger-door control unit (DCU) was replaced; door cycling tests were normal and the fault was cleared.

**Table 4:** Definition of dependent and independent variables.

Variable	Description	Value/Encoding
Dependent variable		
Delay severity	Ordinal label derived from observed delay duration and major operational interventions	0: no impact (<2 min); 1: mild (2–15 min); 2: moderate (15–30 min); 3: severe (≥30 min or turn-back/passenger clearance triggered)
Independent variables		
Time period	Whether the fault occurred during peak hours	0: off-peak; 1: peak
Station type	Station category at which the fault occurred	0: non-hub; 1: hub
Adjacent to escalator	Whether the fault location is adjacent to an escalator	0: no; 1: yes
Fault component features	Three-level tree-structured taxonomy of door components/failure modes	Tree/path encoding; converted to indicator features and later rendered into text slots

**Table 5:** Hyperparameter settings for retrieval, fine-tuning, and decoding.

Hyperparameter	Meaning	Source	Value
α	Mixing weight between dense retrieval and BM25 in hybrid scoring.	Equation (19)	0.6
$k_{1}$	BM25 term-frequency saturation parameter.	Equation (18)	1.2
*b*	BM25 length-normalization parameter.	Equation (18)	0.75
$K_{0}$	Size of the initial candidate pool before reranking.	–	30
*k*	Number of top reranked chunks retained for evidence selection.	Equation (20)	10
$B_{\max}$	Maximum token budget for packed evidence context.	Equation (22)	2000
β	Relevance–diversity trade-off coefficient in MMR-based evidence selection.	Equation (22)	0.7
τ	Temperature in the InfoNCE loss for dense retriever training.	Equation (21)	$5.0\times 10^{-2}$
*r*	LoRA rank for parameter-efficient supervised fine-tuning.	–	8
$\alpha_{\mathrm{LoRA}}$	LoRA scaling factor.	–	32
$\lambda_{\mathrm{sch}}$	Weight of schema violation loss.	Equation (15)	0.5
$\lambda_{\mathrm{role}}$	Weight of role prediction loss.	Equation (15)	0.5
$\lambda_{\mathrm{cite}}$	Weight of citation grounding loss.	Equation (15)	1.0
$\lambda_{\mathrm{reg}}$	Weight of $l_{2}$ regularization.	Equation (15)	$1.0\times 10^{-5}$
Learning rate	Optimizer learning rate for AdamW.	–	$1.0\times 10^{-4}$
Batch size	Mini-batch size used in supervised fine-tuning.	–	16
Epochs	Number of fine-tuning epochs.	–	5
μ	Penalty coefficient for compliance-aware decoding.	Equation (27)	2.0
ρ	Threshold for step-level evidence support.	Equation (28)	0.7
Beam width	Beam size used in beam search decoding.	–	4
γ	Margin parameter in the KG embedding loss.	Equation (24)	1.0
λ	Restart probability in personalized propagation over the KG.	Equation (25)	0.15

**Table 6:** Retrieval performance under different evidence selection settings.

Setting	Relevance (Recall@5 ↑)	Compactness (FirstHit/*B* ↓)	Verifiability (Coverage@*B* ↑)
LangChain	0.68	0.64	0.57
Dense-only ($s_{\mathrm{dense}}$)	0.63	0.71	0.58
BM25-only ($s_{\mathrm{BM}25}$)	0.55	0.62	0.54
Hybrid ($s_{\mathrm{hyb}}$)	0.71	0.55	0.66
Hybrid + rerank ($s_{\mathrm{hyb}}$ + cross-encoder)	0.78	0.46	0.71

*Note:* ↑ indicates that a higher value is better; ↓ indicates that a lower value is better.

**Table 7:** EOS generation quality under different fine-tuning settings.

Setting	SchemaPass	RoleAcc	CiteCov	UsableAns (95% CI)
B0: Pure LLM API (no RAG, no FT)	0.28	0.35	-	0.15 [0.10, 0.21]
B1: Pure LLM API + RAG (prompted, no FT)	0.47	0.52	0.40	0.26 [0.20, 0.33]
B2: SFT-NLL (FT, no structured losses)	0.58	0.69	0.42	0.41 [0.34, 0.49]
B3: +Schema+Role (FT + $L_{\mathrm{sch}}+L_{\mathrm{role}}$)	0.81	0.83	0.56	0.66 [0.58, 0.73]
B4: Full (FT + $L_{\mathrm{sch}}+L_{\mathrm{role}}+L_{\mathrm{cite}}$)	0.88	0.91	0.73	0.83 [0.77, 0.88]

**Table 8:** Example of input prompt and generated EOS for Scenario 1: on-site emergency response.

Structured Question (Input)	Sample Output Result (EOS)
**[Instruction]:** You are an expert in urban rail transit emergency response. Based on the following fault operational context and retrieved maintenance regulations, generate a standardized Emergency Operation Scheme (EOS). **[Contextual Features]:** **Time Period:** Peak hours (08:18) **Location:** Line 6, Yuanshen Sports Center Station (up direction) **Train ID:** AC19-0640 (Type: 06C03) **Fault:** Door 1-1 failure; the door fails to close/lock. **[Retrieved Evidence]:** Chunk 1: “During peak hours, if a door fault cannot be rectified within 60 s, immediately isolate *…*” Chunk *n*: *…*	**[Generated EOS]:** **Phase 1: Initial Assessment** **{Driver}** Report the fault location (Car 1, Door 1) to the OCC and confirm the “Door Not Locked” status on the HMI. **Phase 2: Emergency Handling** **{Driver}** Visually check for anti-clamp obstacles. **{Driver}** Use the square key to mechanically isolate (cut out) the door and confirm that the mechanical lock is “Locked”. **{Driver}** Verify that the HMI status changes from “Red” to “Blue/Yellow” (Isolated). **Phase 3: Service Restoration** **{Driver}** Resume operation under signal authority.

**Table 9:** Example of input prompt and generated MOS for Scenario 2: depot maintenance.

Structured Question (Input)	Sample Output Result (MOS)
**[Instruction]:** You are an expert in urban rail transit maintenance. Based on the following fault diagnosis and retrieved technical manuals, generate a standardized Maintenance Operation Scheme (MOS). **[Contextual Features]:** **Time Period:** Maintenance window (depot) **Train ID:** AC16-1127 (Type: 11A01) **Diagnosis:** Limit switch (S1/S4) logic error **Impact:** No delay; maintenance required **[Retrieved Evidence]:** Chunk 1: “If the S1 signal fluctuates, replace the switch component.” Chunk 2: “After replacement, perform a door cycle test to verify stable operation.”	**[Generated MOS]:** **Phase 1: Diagnosis & Preparation** **{Maintenance Staff}** Download the fault log and confirm the S1/S4 switch codes. **{Maintenance Staff}** Isolate the power supply for safety. **Phase 2: Component Replacement** **{Maintenance Staff}** Remove the cover plate. **{Maintenance Staff}** Replace the faulty S1/S4 switches (ZCD21). **{Maintenance Staff}** Adjust the switch gap. **Phase 3: Verification & Closure** **{Maintenance Staff}** Restore power and perform a “Door Cycle Test” (20 cycles). **{Quality Inspector}** Verify the signal logic. **{Maintenance Staff}** Close the work order and tag the removed parts.

## Data Availability

The data will be available on request.

## Supplementary Materials

The following supporting information can be downloaded at: https://www.mdpi.com/article/10.3390/s26062006/s1.

## Abbreviations

The following abbreviations are used in this manuscript:

API	Application programming interface
BM25	Best Matching 25 (Okapi BM25)
BN	Bayesian network
DCU	Door control unit
DFMIS	Dispatching Fault Management and Analysis Database System
DDU	Door display unit
EDCU	Electronic door control unit
EOS	Emergency operation scheme
FTA	Fault tree analysis
HMI	Human–machine interface
KG	Knowledge graph
LLM	Large language model
LoRA	Low-rank adaptation
LSTM	Long short-term memory
MMR	Maximum marginal relevance
MOID	Metro operation incident database
MOS	Maintenance operation scheme
OCC	Operations Control Center
RAG	Retrieval-augmented generation
SOP	Standard operating procedure
URT	Urban rail transit
